# 3D-Printed Tumor-on-Chip for the Culture of Colorectal Cancer Microspheres: Mass Transport Characterization and Anti-Cancer Drug Assays

**DOI:** 10.3390/bioengineering10050554

**Published:** 2023-05-05

**Authors:** Mónica Gabriela Sánchez-Salazar, Regina Crespo-López Oliver, Sofía Ramos-Meizoso, Valeri Sofía Jerezano-Flores, Salvador Gallegos-Martínez, Edna Johana Bolívar-Monsalve, Carlos Fernando Ceballos-González, Grissel Trujillo-de Santiago, Mario Moisés Álvarez

**Affiliations:** 1 Centro de Biotecnología-FEMSA, Tecnológico de Monterrey, Campus Monterrey, Monterrey 64849, Mexico; monica.sanchez@tec.mx (M.G.S.-S.); salvador.gallegos@tec.mx (S.G.-M.);; 2 Departamento de Mecatrónica e Ingeniería Eléctrica, Escuela de Ingeniería y Ciencias, Tecnológico de Monterrey, Monterrey 64849, Mexico

**Keywords:** cancer-on-chip, tumor-on-chip, colon cancer, microsphere, continuous flow, mass transport, drug assay

## Abstract

Tumor-on-chips have become an effective resource in cancer research. However, their widespread use remains limited due to issues related to their practicality in fabrication and use. To address some of these limitations, we introduce a 3D-printed chip, which is large enough to host ~1 cm^3^ of tissue and fosters well-mixed conditions in the liquid niche, while still enabling the formation of the concentration profiles that occur in real tissues due to diffusive transport. We compared the mass transport performance in its rhomboidal culture chamber when empty, when filled with GelMA/alginate hydrogel microbeads, or when occupied with a monolithic piece of hydrogel with a central channel, allowing communication between the inlet and outlet. We show that our chip filled with hydrogel microspheres in the culture chamber promotes adequate mixing and enhanced distribution of culture media. In proof-of-concept pharmacological assays, we biofabricated hydrogel microspheres containing embedded Caco2 cells, which developed into microtumors. Microtumors cultured in the device developed throughout the 10-day culture showing >75% of viability. Microtumors subjected to 5-fluorouracil treatment displayed <20% cell survival and lower VEGF-A and E-cadherin expression than untreated controls. Overall, our tumor-on-chip device proved suitable for studying cancer biology and performing drug response assays.

## 1. Introduction

Monolayer cell culture continues to be the model most frequently used in fundamental cancer research and anti-cancer drug discovery [1]. However, these models lack the three-dimensional (3D) architecture of natural tissues and therefore fail to recreate the dynamics of human physiology (i.e., the presence of flows, transport, and concentration gradients) [1,2,3,4]. More than half of all potential chemotherapeutic drugs fail during phases II and III of their clinical trials because, at this time, the in vitro and in vivo screening models so poorly recapitulate cancer tumors in humans [5]. Among the vast array of experimental treatments tested in monolayer models, only 4% of new chemotherapeutic drugs are granted FDA approval [6]. Therefore, establishing systems that enable efficient drug screening under conditions that closely simulate the biology and physiology of cancer, while also reflecting the dynamic nature of tumors and their interaction with their surroundings, remains a pressing challenge [7].

One approach that could meet this challenge is to use tumor-on-chips [2,7,8] (ToCs), which are microfluidic devices used to culture cancerous microtissues. ToCs integrate key elements, such as the 3D architecture of real tumors [9] and the possibility of recreating the dynamics that occur in the tumor niche (i.e., convective [10] and/or diffusive transport), making them promising new tools for enhancing the efficacy of cancer research. 

Experimental evidence is now showing that the response of cancerous tissue to drugs differs under static or dynamic conditions [11]. 

Multiple examples of TOCs have been published, and recent reviews accurately describe the state of the art in this field [2,12,13,14]. However, currently available ToCs still exhibit important limitations that restrict their wider use in cancer research. Some of the existing ToC models are not user friendly and require specialized or highly trained personnel for their proper and reproducible fabrication and/or operation. In addition, most are capable of hosting only very small amounts of tissue, whereas the aspiration of tumor-tissue engineers is to fabricate tumor models that closely recapitulate both the 3D architecture and the size of real tumors [15]. 

In this work, we address three of the limitations that we frequently encounter with cancers-on-chips. These constraints are related to the complexity of assembly and use of the devices, their microscale size, and their inability to foster a homogeneous and well-mixed culture environment. Here, we introduce a simple and easy-to-operate ToC system that can mimic several relevant aspects of the colorectal cancer microenvironment and thereby emulate realistic therapeutic interventions that mimic the intravenous administration of anti-cancer drugs. In pharmacodynamic studies, a typical assumption is that the human body is a well-mixed system; therefore, seeking homogeneity in ToCs aligns well with this assumption. 

The ToC that we present and characterize consists of a 3D-printed device featuring a rhomboidal culture chamber filled with hydrogel microspheres loaded with colon cancer cells. This ToC can be perfused continuously to provide constant nutrient and waste exchange. We envision that this ToC can be used to model different cancer types with various degrees of complexity. In this paper, as a proof of concept, we illustrate its ability to recapitulate a simple colorectal cancer microenvironment. We characterized the transport and mixing performance within the chip and demonstrated its use in a set of anti-cancer drug experiments using 5-FU (5-fluorouracil), a commonly used drug for the treatment of colorectal cancer. Considering the high incidence of colorectal cancer in modern society, the recapitulation of colorectal cancer has received insufficient attention. Colorectal adenocarcinoma, or colorectal cancer, is currently the third leading cause of death related to cancer in adults worldwide [16], and its incidence is expected to increase by 56% between 2020 and 2040, leading to an increase in mortality of about 69% [16]. Thus, the need for relevant colorectal cancer models to enable research efforts in fundamental research and drug screening is evident now and will continue to intensify in the near future. Unfortunately, only a handful of colorectal ToCs have been described in the literature. In 2019, Carvalho et al. developed a PDMS-based microfluidic chip to mimic a vascularized colorectal cancer niche and evaluated the efficacy of anticancer drug delivery from nanoparticles [17]. Strelez et al. described an organ-on-chip model for studying colorectal cancer intravasation. Their model integrates fluid flow, peristalsis movement, and endothelial and epithelial compartments divided by a porous membrane [18]. Habanjar et al. developed a microfluidic device that considered the co-culture of different human cell types in an extracellular matrix [19].

## 2. Materials and Methods

### 2.1. Chip Design and Fabrication

**Figure 1:**
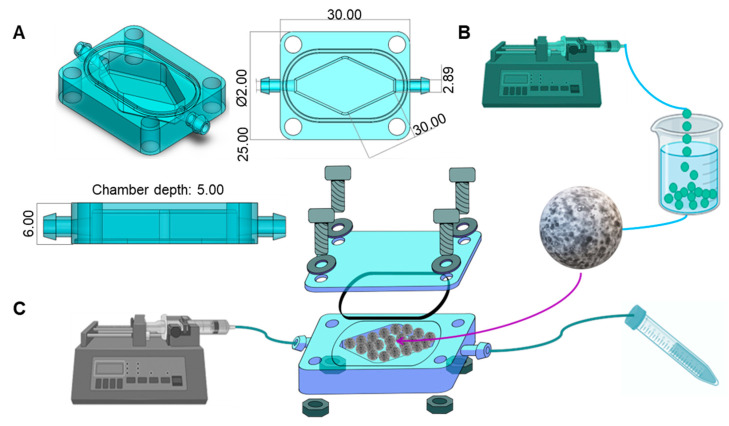
Design and configuration of a colorectal tumor-on-chip (ToC). (**A**) Shape and dimensions (in mm) of the 3D-printed mini-reactor. The chip was designed in SolidWorks. (**B**) Fabrication of Caco2 microtumors in hydrogel microspheres. The bioink consisted of 3.5% alginate, 2.5% GelMA, and Caco2 cells at a density of 1.5 × 10^6^ cells/mL. Microspheres were fabricated by dripping the bioink into a bath of 4% CaCl_2_ and 12 g/L Tween 80. (**C**) Tumor-on-chip system setup. We filled the ToC with Caco2 microspheres, covered it with an acrylic lid, and connected it to a syringe pump and a waste tube to close the system.

We designed a simple and versatile ToC that houses a tumor microtissue and enables the continuous perfusion of culture medium (with or without anti-cancer drugs). The ToC consists of a 3D-printed rhomboid culture chamber embedded within a rectangular solid structure, with inlet and outlet ports designed for easy connection to silicon tubing (Figure 1A). 

The rhomboid chamber was filled with hydrogel microbeads (fabrication is described in Microsphere fabrication) (Figure 1B). The chip is built to hold a transparent lid that is secured by screws and nuts, and a rubber O-ring to prevent liquid leaks. This device was designed in SolidWorks and 3D-printed using Clear Resin (FormLabs, Somerville, MA, USA) in a stereolithographic 3D printer (Form 3, FormLabs, Somerville, MA, USA). The use of an acrylic lid and clear resin enables visualization of the interior of the culture chamber. The configuration and dimensions of the chip are detailed in Figure 1A. 

To perfuse culture media, we connected soft transparent tubing (MasterFlex, Zapopan, JAL, México) to the inlet and outlet ports. The inlet tubing was connected to a syringe containing the desired culture medium, while the outlet tubing was connected to a 15 mL tube for the waste medium recollection. The syringe was placed in a syringe pump to provide a continuous flow at 1 μL/min. The complete system setup is shown in Figure 1C.

### 2.2. Microsphere Fabrication

We fabricated hydrogel microbeads composed of a mixture of gelatin methacryloyl (GelMA) [20] and low viscosity alginate (Sigma-Aldrich, St. Louis, MO, USA). GelMA with a high degree of methacrylation was prepared in the laboratory according to our previously published protocols [21]. Briefly, the GelMA pregel was prepared by mixing 5% (*w*/*v*) GelMA dissolved in Dulbecco’s phosphate buffered saline (DPBS) and 0.067% (*w*/*v*) lithium phenyl-2,4,6-trimethylbenzoylphosphinate (LAP) as a photoinitiator for 20 min at 70 °C. The GelMA mixture was filtered through a 0.22 µm filter and stored at 4 °C until use. Low viscosity alginate was prepared in DPBS at a 7% (*w*/*v*) concentration by agitation at 40 °C for 1 h. Hydrogel microbeads without cells were fabricated by mixing 5% GelMA pregel and 7% alginate at a 1:1 ratio (final concentrations of 2.5% and 3.5% for alginate and GelMA, respectively). 

Cell-laden microbeads were prepared by resuspending Caco2 cells in the GelMA/alginate pregel to achieve a final cell density of 1.5 × 10^6^ cells/mL. This suspension was then dripped through a 27 G tip at a flow rate of 3 mL/min using a syringe pump. Drops were received in a 4% calcium chloride solution supplemented with 12 g/L Tween 80 at 4 °C (the calcium served to crosslink the alginate). The distance between the tip and the bath was set to 15 cm to allow the formation of hydrogel microspheres. The spheres were maintained at 4 °C for 5 min and then exposed to UV light (OmniCure S1500, Mississauga, ON, Canada) at 365 nm for 30 s and a distance of 15 cm to photo-crosslink the GelMA. The resulting Caco2-loaded spheres were washed three times with PBS, placed in 6-well culture plates, and cultured in Eagle’s minimum essential medium (EMEM) for 24 h to allow them to stabilize.

### 2.3. FITC-Diffusion Experiment

**Figure 2:**
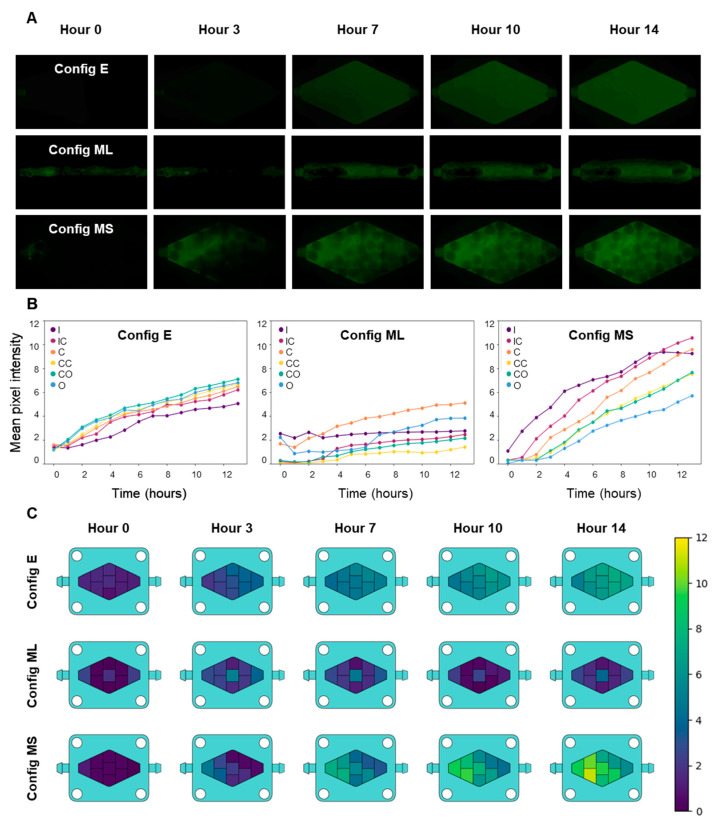
Comparison of culture medium diffusion in three different configurations of the rhomboid chip: Config E, an empty culture chamber; config ML, a monolithic 3D GelMA hydrogel (filling the chamber); and config MS, hydrogel microspheres filling the chamber. (**A**) Diffusive/convective transport of FITC-dextran inside the chip culture chamber in the three different configurations. (**B**) Quantification and comparison of culture medium diffusion in the three different settings, as represented by the increase in fluorescence intensity (FITC-dextran), measured by mean pixel intensity. I: inlet, IC: inlet-center, C: center, CC: center corners, CO: central-outlet, O: outlet. (**C**) Heatmaps representing the mean concentrations of a tracer throughout the chip in different zones at several time points, as estimated by the increase in fluorescence in the FITC-dextran experiments.

We characterized the efficiency of the convective/diffusive transport within the rhomboidal chips using FITC-dextran dissolved in EMEM. To do this, we continuously pumped EMEM containing 0.1 mg/mL FITC-dextran (40 kDa) (Sigma-Aldrich, St. Louis, MO, USA) through the chips at a flow rate of 1 µL/min. EMEM was used to provide conditions similar to those used during the on-chip culture (Figure 2A). The FITC-diffusion experiments were conducted to compare three variations of the rhomboidal chip configurations that differed in the culture chamber filling: (1) a chip without any hydrogel constructs in the culture chamber (referred as config E); (2) a chip filled with a 5% GelMA monolythic hydrogel casted on the culture chamber and featuring a central hollow channel connecting the inlet and the outlet (referred as config ML); and (3) a chip filled with GelMA/alginate microspheres (referred as config MS). In preliminary experiments, we also compared the diffusive transport among chips with rectangular vs. rhomboidal culture chambers and filled with hydrogel microspheres. 

Diffusion experiments were conducted under an inverted microscope; the microscope was configured to capture frames of the culture chamber under fluorescent LED illumination (FITC channel) every half hour for 14 h in overnight experiments. All pictures were taken at a 28.2% LED intensity and 30 ms exposure time. 

Time series consisting of images taken each hour beginning at hour 0 (14 pictures total) were analyzed using ImageJ software (NIH, Bethesda, MD, USA) without color correction of the FITC fluorescence. To do this, we obtained the average fluorescence intensity at nine different regions of the rhomboid chamber (Figure S1) by considering five squares 45,000 pixels in area. Similarly, we divided the culture chamber of the rectangular chip into regions (Figure S2). Then, using ImageJ analysis, we obtained the mean gray value in these five samples per area every hour. These samples were then grouped together throughout all the independent repeats of an experiment (N = 5) in a single variable per area using in-house software written in the Python programming language (Wilmington, DE, USA). This procedure leads to a 5 × 14 matrix (number of experiments × the number of hours per experiment). From this matrix, a mean gray value is obtained every hour to produce the graphs and heat maps shown in this work. Due to symmetry considerations, the intensity calculated in the analogous regions of the chip above and below the central axis was averaged to calculate and plot only six fluorescence profiles. The regions that were averaged were the top inlet & bottom inlet (to calculate the inlet-center value [IC]); the top corner and bottom corner (to calculate the center-corner value [CC]); and the top outlet and bottom outlet (to calculate the center-outlet value [CO]). We ultimately reported the average fluorescence value for six regions in each chip configuration, namely, the inlet (I), inlet-center(IC), center corners (CC), center (C), center-outlet (CO), and outlet (O). The average pixel intensity for each hour of fluorescence was used to plot the evolution of the fluorescence in each region. Heatmaps were further developed using Python programming and picture editing software (Clip Studio Paint, Tokyo, Japan; Procreate, Tasmania, Australia).

### 2.4. Cell Lines and Cell Culture

The Caco2 cell line was purchased from ATCC and were cultured in EMEM (Sigma-Aldrich, St. Louis, MO, USA) supplemented with 10% fetal bovine serum (FBS) and 1% penicillin–streptomycin. Cells were incubated at 37 °C, in 5% CO_2_ atmosphere and 100% humidity, for maintenance culture and for experiments. For passaging, cells were detached with 0.25% trypsin/0.53 mM EDTA, and a 0.4% trypan blue solution was used (in 1:1 ratio) for counting and estimation of cell viability.

### 2.5. Chip Assembly

We sterilized the chips by immersion in 70% ethanol and UV light exposure for 15 min. The acrylic lids were cleaned with Lysol and sterilized under UV light for 15 min. The hoses, waste tubes, screws, and nuts were sterilized in an autoclave for 15 min at 121 °C. The Caco2 microspheres, previously fabricated in 24-well plates, were retrieved from the culture plates using a sterile spatula and placed in the chip culture chamber. We then placed and secured the lids with screws and nuts and connected a syringe with culture medium to the inlet and a waste tube to the outlet using sterile tubing (MasterFlex 96400-14, Zapopan, JAL, México). We then filled the reservoirs with culture medium, loaded the syringes into a syringe pump (IPS-14RS, Inovenso, Boston, MA, USA), placed the chip and the pump inside the incubator, and set the perfusion flow rate at 1 µL/min.

### 2.6. Glucose Consumption Measurements

We measured the outlet glucose concentration every 24–48 h during the 10 days of each culture experiment using a commercial glucometer (Accu-Chek, Roche, Switzerland) and glucose strips (Accu-Chek Active, Roche, Switzerland). The glucose concentration was reported as mg/dL, which we converted to mg/mL. Daily glucose consumption was calculated based on the difference between the glucose concentrations at the inlet and the outlet of the chip and considering the flow rate.

### 2.7. Live/Dead Analysis

The cell viability of the on-chip cultures was assessed using Live/dead staining performed with a LIVE/DEAD™ Cell Imaging Kit (ThermoFisher, USA) according to the manufacturer’s instructions. We obtained fluorescence images using an Axio Observer.Z1 microscope (Zeiss, Oberkochen, Germany). Reconstructed micrographs were acquired with the microscope software (Zen Blue Edition, Zeiss, Oberkochen, Germany). Live/dead images were analyzed using ImageJ software (NIH, Bethesda, MD, USA) by measuring the fraction of the area occupied by live cells (i.e., cells stained green) or dead cells (i.e., cells stained red) in randomly selected microspheres in each experiment. For each experimental image, the mean area was obtained, and then the percentage of live and dead cells was calculated.

### 2.8. In Vitro Drug Assessments

We assessed the response of Caco2-loaded microspheres to treatment with 10 mM 5-fluorouracil (5-FU). We first filled Caco2-loaded beads into the chip culture chambers and perfused them with EMEM for 5 days. After this on-chip stabilization period, we perfused the chips with EMEM + 10 mM 5-fluorouracil (5-FU) for 46 h to emulate an intravenous dosage of 5-FU chemotherapy. We then changed the perfusion back to EMEM alone for the remaining three days of the experiment. We performed live/dead staining and qPCR assays on the Caco2 microbeads to evaluate survival and relevant marker expression at the end of the experiment.

### 2.9. RT-qPCR

The expression of relevant Caco2 genes was evaluated by first washing the GelMA-alginate spheres with PBS 1X (Sigma-Aldrich, St. Louis, MO, USA) and then digesting the spheres with 30–50 μL proteinase K (Omega Bio-tek, Norcross, GA, USA) in a water bath at 50 °C for 2 h, with intermittent vortexing. The samples were then centrifuged at 10,000 RPM for 5 min, and the supernatant was recovered. The RNA extraction was conducted using the RNAeasy Mini Kit (QIAGEN, Germantown, MD, USA) according to the manufacturer’s instructions. Total RNA was quantified using Nanodrop (ThermoFisher, Waltham, MA, USA), considering a 260/280 ratio of 2.0. For gene expression, we used an RT-qPCR SYBR Green kit (QIAGEN, Germantown, MD, USA) according to the manufacturer’s instructions. RT-qPCR was conducted using a Rotor gene SYBR green filter (QIAGEN, Germantown, MD, USA). GAPDH was used as a housekeeping gene to estimate the relative gene expression of the vascular endothelial growth factor (VEGF-A) and E-cadherin (E-cad) using 2^−∆∆Ct^ method. One-way ANOVA, followed by the post hoc Bonferroni test (*p* values < 0.05), was used to estimate statistical differences. The primer sequences used in these RT-qPCR experiments are listed in Table 1.

### 2.10. Immunostaining

We performed immunostaining assays according to a previously published protocol [22] with some modifications. Briefly, after 10 days of culture, we retrieved the microspheres from the chip, embedded them in a solution of 7% gelatin + 10% sucrose prepared in PBS, cut them into sections, and washed them twice with PBS for 15 min to remove the remnant cell culture media. We then immersed the constructs in 4% paraformaldehyde for 30 min to fix the cells and washed them three times with PBS. We then permeabilized the constructs for 45 min with 0.5% Triton X-100 in PBS. We blocked non-specific adhesion sites using a blocking solution of 5% bovine serum albumin (BSA), 0.3 M glycine, and 0.5% Triton X-100 in PBS with overnight incubation at 4 °C. We prepared primary antibodies for different targets (anti-VEGF, anti-E-cad) in a 1:100 dilution in the blocking solution, added them to the constructs, and incubated them overnight at 4 °C. We then washed the constructs three times with a washing solution of 0.2% Triton X-100 and 0.04% Tween 20 in PBS and incubated them in the dark with a secondary antibody (donkey anti-mouse IgG H&L conjugated with Alexa Fluor 594, ab150108, Abcam, Boston, MA, USA) overnight at 4 °C. Cell nuclei and cytoskeletons were counterstained by immersion in 3 µg/mL DAPI (Sigma-Aldrich, St. Louis, MO, USA) and 3 µg/mL phalloidin (Abcam, Boston, MA, USA) in the washing solution for 1 h at 37 °C.

### 2.11. Imaging and Analysis

Bright field and fluorescence images were obtained using an Axio Observer.Z1 microscope (Zeiss, Oberkochen, Germany) equipped with Colibri.2 LED illumination and an Apotome.2 system (Zeiss, Oberkochen, Germany). Reconstructed micrographs were acquired using the microscope software (Zen Blue Edition, Zeiss, Oberkochen, Germany). We analyzed the images with ImageJ (NIH, Bethesda, MD, USA). For live/dead quantification, ImageJ (NIH, Bethesda, MD, USA) was used to determine the fraction of red and green stained areas with respect to the total projected area covered by cells in each microscope image.

### 2.12. Statistical Analysis

We used the *t*-test to estimate statistical differences between pairs of treatments. A *p* value < 0.05 was considered statistically significant.

## 3. Results and Discussion

### 3.1. Rationale of the Design

In this study, we introduced and characterized a millifluidic device that addressed three frequently encountered limitations of ToCs related to their complicated assembly and use, small size, and inability to foster a homogeneous and well-mixed environment. ToCs are often based on the superposition of several layers of PDMS and/or PMMA, which requires that these chips be assembled on a one-by-one basis. These ToCs are also more prone to leakage and contamination events. In addition, the chips typically can host only a limited amount of cancerous tissue; therefore, these ToCs are based on the culture of a few avascular micro-spheroids in the size range of 20–400 µm [23,24]. Our aim in this work was to develop a flexible and cost-effective mini-reaction platform capable of hosting simple (and not so simple) microtissues without depending on sophisticated and costly peripherals and control systems. Thus, we have introduced a versatile chip that consists of a 3D-printed rectangular block containing a rhomboidal culture chamber 0.948 cm^3^ in size and equipped with inlet and outlet ports and a transparent acrylic lid held in place with screws and nuts. The inlet and outlet were connected to a syringe containing culture medium and a waste tube, respectively. Culture medium was perfused at a flow rate of 1 μL/min using a syringe pump (Figure 1). 

The design of organ-on-chip systems also rarely considers the requirement for good mixing conditions to favor homogeneous culture microenvironments within the device. The experiments conducted here showed that both the rhomboidal shape of the culture chamber and the spherical hydrogel microbeads filling the culture chamber promoted adequate mixing and recreation of dynamic conditions that fostered adequate tissue growth within the microbeads. The 3D design of the chip was easily customized using SolidWorks software and was printable using a commercially available 3D printer. We anticipate that these features will greatly facilitate the widespread use of this cancer-on-chip device. Our 3D-printed device is suitable for hosting any type of 3D tissue model (e.g., cells embedded in hydrogel fibers or beads), and it can withstand a wide range of culture medium flows. The geometry of the chamber also provides a user-friendly setup and operation, and the clear resin and acrylic lid enabled easy visualization of the microtissues hosted within the culture chamber.

### 3.2. On-Chip Mixing and Transport Characterization

The aim of ToCs is to recapitulate the dynamic environment of the tumor niche more faithfully than is possible with static systems [11]. To achieve this, the integration of convective and diffusive transport in ToCs is relevant [9,11,23,25], since tumors are dynamic entities that receive nutrients and drugs through a combination of convective and diffusive transport. Nutrients and drugs, dissolved in the blood, are first convectively transported to tissues by arterial capillaries, from which they then diffuse through distances ranging from 10 to 100 µm within tissues [26] to finally reaching the tumor cells. Thus, assessing the adequacy of both mixing and diffusive transport is critical for ToCs. Importantly, the geometry and configuration of the chip determine mixing and transport. In the literature, rectangular [1,18,23], circular [6,27,28], and rhomboidal [9,29] shapes are frequently used for the fabrication of ToCs.

In a preliminary set of experiments, we analyzed mixing and diffusion in chips that featured rectangular or rhomboidal culture chambers by perfusing a FITC-dextran solution while monitoring the dispersion of the fluorescent dye using a microscope. We observed a more even distribution of fluorescence in the rhomboid than in the rectangular chip since all the zones were reached by the FITC-dextran (Figure S2). In addition, these tracer experiments revealed that all corners in the rectangular configuration, particularly the corners located closer to the outlet, acted as “dead” zones, where the fluorescence increase was much slower than in the central portions of the device. 

We also compared mixing and diffusive transport between three different rhomboidal chip embodiments. In a first configuration of the chip (referred as E in Figure 2), the culture chamber was empty (did not contain any hydrogel). In a second configuration (referred as ML), a solid block of GelMA was casted inside the rhomboidal chamber, and a central hollow channel was fabricated to allow communication between the chip inlet and outlet. In the third configuration of the chip (referred as MS), the culture chamber was filled with hydrogel microspheres (Figure 2A). The transport conditions in each of these systems differed drastically. Figure 2A compares the fluorescence results for the three systems. 

The process of dispersion and mixing of the fluorescent tracer seemed faster in the rhomboidal device that contained microspheres, and the fluorescence intensity was also the greatest. In the absence of any hydrogel construct in the culture device, the dispersion of FITC-dextran (Figure 2A) was slower than in the chip filled with microspheres, because transport was limited by diffusion from the central region to the lateral regions of the chip. In the device containing hydrogel microbeads, the spheres act as deflectors that enhance mixing and promote homogeneity among the different regions of the culture chamber in shorter times. The device that contained the channeled block of hydrogel did not reach a homogeneous and steady distribution of the tracer even after 14 h, and the fluorescence seemed less homogeneous than in the other two configurations. This observation is relevant since one of the most common configurations of ToC systems is solid blocks of ECM-like hydrogels loaded with cancer cells [1,23,30]. While the channeled block configuration may be useful in microscale studies where diffusive distances are typically small (µm scale), this geometry may become unsuitable in larger ToCs in which transport limitations may lead to nutrient starvation [1].

We also quantified the fluorescence signals in six different locations (Supplementary Figure S1) within the culture chamber of each chip using image analysis techniques. Figure 2B shows the fluorescence measurements derived from the images obtained from different locations in the three different rhomboidal chip configurations analyzed. The results of this quantitative analysis are consistent with the qualitative observations already discussed. For instance, the fluorescence signal increased in a more uniform manner in the chip that contained alginate-GelMA spheres than in the other two configurations (Figure 2B,C). Image analysis also confirmed that the fluorescence was distributed most evenly and rapidly in the chip configuration without hydrogel. The configuration consisting of a monolithic piece of hydrogel with a central channel showed fluorescence increases mainly restricted to the area surrounding the hollow channel, while the rest of the hydrogel remained a poorly mixed zone. 

The quantitative image analysis also provided additional information. For example, the fluorescence profiles over time showed that the fluorescence distribution was more homogeneous in the empty chamber configuration and that the highest fluorescence intensity occurred in the central-outlet region. The empty chamber configuration exhibited a lower steady-state (asymptotic) fluorescence signal than the configuration containing hydrogels microbeads in the culture chamber. In the microsphere system, the region that accumulated the most fluorescence was the inlet-central region, possibly due to the absorption of the tracer by the hydrogel. Finally, the GelMA system displayed the most heterogeneous diffusive transport, where the central region accumulated the most fluorescence, in agreement with the position of the hollow channel.

We used the fluorescence images and image analysis techniques to develop heatmaps that described the diffusive transport within the three systems (Figure 2C). The fluorescence was greater in the microsphere system than in any of the other systems after 7 h. In general, the system with the best diffusive transport for the purpose of a ToC system was the microsphere system.

### 3.3. Caco2 Microtumor Survival and Development

We used a simple and efficient technique to fabricate Caco2 cell-laden hydrogel microbeads constructed from a mix of alginate and GelMA, polymers commonly used in 3D tissue engineering, with the aim of developing a simple but relevant colon ToC model. The biocompatibility and suitability of this hydrogel combination to support cell growth has been confirmed elsewhere [22,31]. Alginate provides structural stability but with suitable stiffness to still allow cell proliferation, while GelMA provides cell attachment sites [20,32]. In principle, the use of GelMA with different degrees of methacrylation enables tuning the stiffness of the resulting hydrogel, among other preparation variables [33]. For example, we chose GelMA with a high degree of methacrylation to mimic the elastic modulus of the human colon (5.18 MPa) [34].

**Figure 3:**
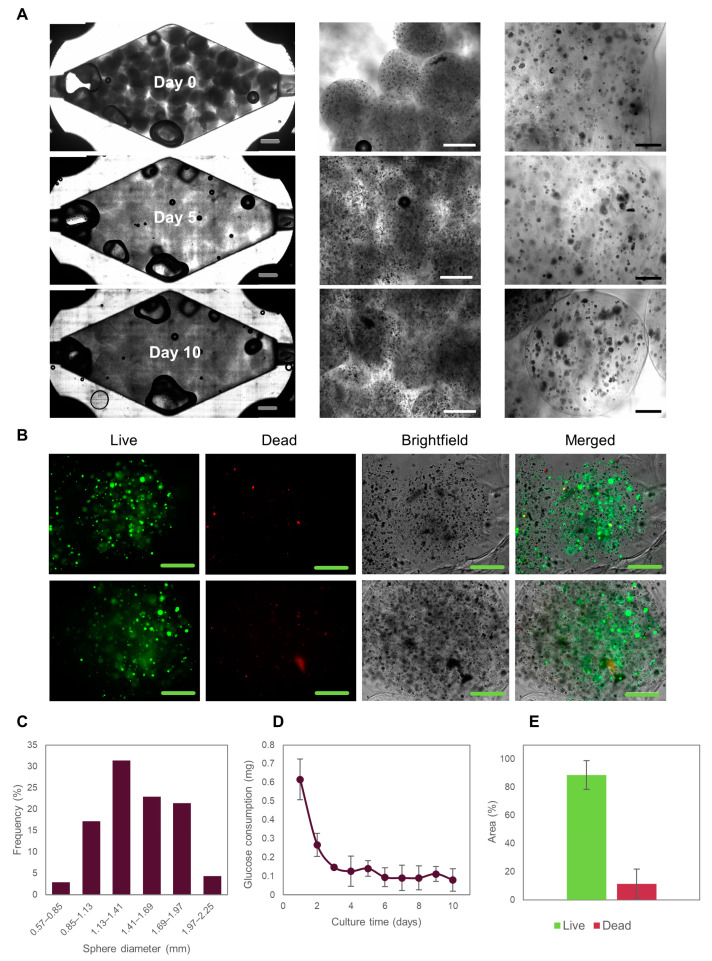
Evolution of Caco2 microtumors embedded in Alg/GelMA inside a rhomboidal 3D-printed tumor-on-chip (ToC). (**A**) Brightfield images of Caco2 microtumors evolution in the ToC. Grey scale bar: 2000 μm. White scale bar: 1000 μm. Black scale bar: 200 μm. (**B**) Live/dead staining of Caco2 microtumors after 10 days of culture in the ToC. Green scale bar: 500 μm. (**C**) Alg/GelMA microspheres size distribution. (**D**) Glucose consumption of Caco2 microtumors cultured in the ToC. (**E**) Live/dead area measurements of Caco2 microtumors.

Caco2 cells embedded within the alginate/GelMA microspheres proliferated and formed multicellular aggregates (Figure 3A,B) that we subsequently refer to as microtumors. This observation is consistent with previous reports [35] in which collagen or gelatin-based hydrogels have been used to encapsulate Caco2 cells [23]. We chose a cell density of 1.5 × 10^6^ cells mL^−1^ to provide sufficient space for cells to grow and form microtumors, while still allowing them to interact through biochemical signaling. Most spheres had diameters between 1.1 and 1.7 mm and a normal size distribution (Figure 3C). As already discussed, hydrogel microspheres served as baffles that deflected the convective flow and enhanced mixing. The transport conditions that prevailed on-chip were sufficient to provide a suitable environment for Caco2 cells, as observed by glucose consumption (Figure 3D). 

Since glucose is the main carbon source in mammalian cells, glucose consumption should be considered a relevant and integral indicator of the metabolic activity of the cells in culture. Glucose consumption stabilized after 5 days of on-chip culture and did not change throughout the 10 days of on-chip culture, suggesting that a metabolic steady-state has been achieved in the microtissues. Achieving a steady state is a highly desirable condition for on-chip cultures [36,37,38] and represents an ideal condition for pharmacological testing (among other studies).

Notably, our device allowed the retrieval of the Caco2 cell-loaded microspheres to facilitate later biological characterization [1]. After 10 days of culture, cell survival was greater than 75%, as determined by live/dead staining and quantification using ImageJ (Figure 3E). The large size of the microspheres provided slow nutrient penetration [1], which is expected to initiate oxygen and nutrient concentration gradients. Indeed, larger microtumors were formed by the cells located on the outer part of the microspheres than at the center of the spheres. Consistently, we observed that most of the dead cells were located in the central region of the microspheres. In general, we did not observe significant differences in cell survival rates in the different zones of the device (Figure S3). Taken together, these results suggest that our system provides a suitable environment for culturing colorectal microtumors for studying cancer biology and performing drug screening for drug development or personalized medicine.

In particular, colorectal cancer-on-chip models have focused on studying cancer development, progression, and cell interactions [1,18], and only a handful of them have assessed drug responses [23,25,29]. Carvalho et al. used a 3D microfluidic chip made of PDMS to evaluate the efficacy of nanoparticle-based drug delivery [17]. Strelez et al. described an organ-on-chip model to study colorectal cancer intravasation; their model integrated fluid flow, peristalsis movement, and endothelial and epithelial compartments divided by a porous membrane [18]. These reports illustrate the importance of re-creating the dynamic environment of the tumor niche to study relevant aspects of the biology of colorectal cancer and to evaluate colorectal cancer therapies. The ToC that we have introduced here offers a simpler approach to the reconstruction of a colorectal cancer niche and supports larger volumes of cancerous tissue that can be interrogated while providing convective and diffusive transport of nutrients and drugs.

### 3.4. Suitability of the Tumor-on-Chip System for Drug Testing

In an additional set of experiments, we used our ToC system to simulate typical colorectal chemotherapy. We perfused 5-FU for 46 h, the same time that it is administered in human colorectal cancer patients treated with chemotherapy [39,40,41,42,43]. 5-FU is a widely used drug in cancer treatment, especially colorectal cancer. The 5-FU anti-cancer effect is attributed to the inhibition of the thymidylate synthase enzyme and the incorporation of its metabolites into both RNA and DNA [44]. This is believed to severely disrupt DNA synthesis, causing DNA strand breaks and cell death, as well as inhibition of the maturation of pre-mRNA and disruption of post-translational modification [44]. 

**Figure 4:**
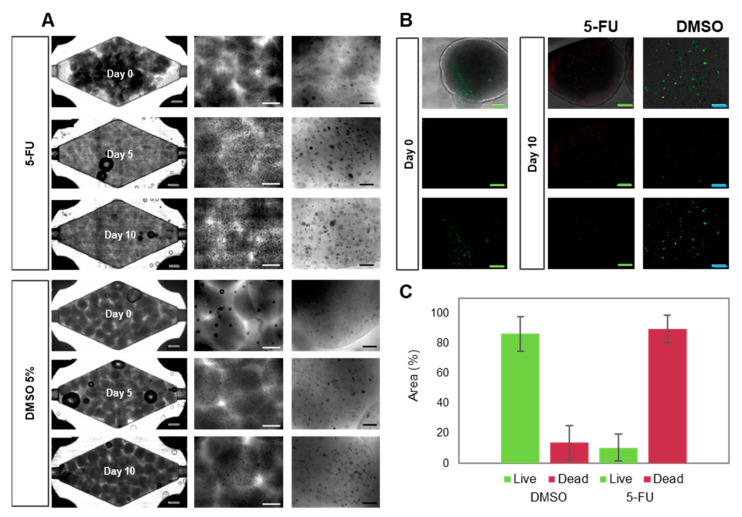
Evolution of Caco2 microtumors embedded in alginate/GelMA before and after on-chip 5-FU exposure. Grey scale bar: 2000 μm. White scale bar: 1000 μm. Black scale bar: 200 μm. Green scale bar: 1000 μm. Blue scale bar: 200 μm. (**A**) Brightfield images of the evolution of Caco2 microtumors with 5-FU (top) or DMSO (vehicle control, bottom) on-chip. (**B**) Representative images of Live/dead staining of Caco2 microtumors as observed at day 0 and day 10 of on-chip culture. (**C**) Determination of Live/dead projected area in Caco2 microtumors treated with 5-FU or DMSO, as calculated by image analysis.

For our 5-FU drug assays, we first cultured Caco2 cell-laden microspheres in the culture chamber of the chip for five days to allow them to reach a metabolic steady-state (Figure 3D). We then initiated the perfusion of 10 mM 5-FU in EMEM culture medium, setting the flow rate of the perfusion system (i.e., the syringe pumps) to simulate a residence time of 46 h in the bloodstream. After 46 h of drug exposure, the Caco2 spheres were perfused with pristine EMEM for three additional days to observe the effects after stopping drug administration. Our ToC system successfully allowed the observation of microtumor drug responses for several days before and after drug administration (Figure 4A). 

We observed that the administration of 5-FU prevented cell proliferation (Figure 4A) and microtumor development. Most cells were unable to form new aggregates during 5-FU treatment. Also, the microtumors that formed during the stabilization period decreased in size during treatment. Live/dead images showed significantly higher cell decay than was observed in perfusion experiments with pristine culture media or DMSO (Figure 4B). Quantification of dead cells revealed an average cell survival below 20% (Figure 4C). 

Alternative feeding regimes have been previously tested by other research groups [25,45]. For example, Kwapiszewska et al. fabricated solid cancer spheroids and then transferred them to a tumor-on-chip device to expose them to a continuous dose of 0.125–1 mM 5-FU for 15 min, followed by culture for 24 h in static conditions after drug administration [45]. In another report, Skardal et al. cultured colon cancer constructs for 14 days and later incubated them with 0, 1, 10, and 100 mM 5-FU doses for 24 h. They then measured mitochondrial metabolism and observed a decrease corresponding to increasing doses of 5-FU [25]. 

**Figure 5:**
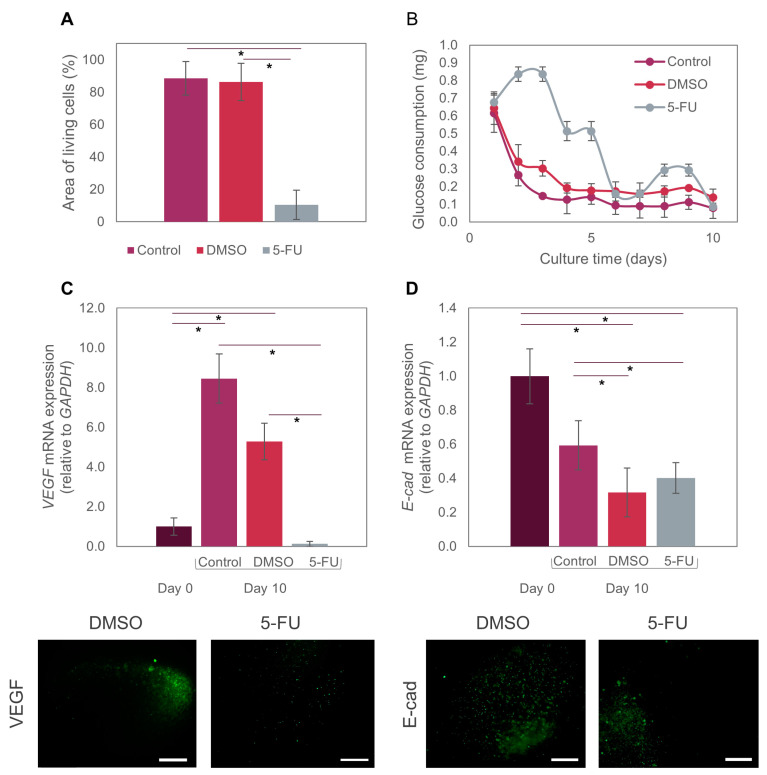
Effect of 5-FU on Caco2 microtumors. (**A**) Comparison of Live/dead area measurements of Caco2 microtumors exposed to the different treatments. (**B**) Glucose consumption by Caco2 microtumors treated with 5-FU on-chip. (**C**) Differential expression of VEGF-A at day 0 and day 10 in microtumors exposed to different treatments (control, DMSO, 5-FU) on-chip. (**D**) Differential expression of E-cad at day 0 and day 10 in microtumors exposed to different treatments (control, DMSO, 5-FU) on-chip. We used the *t*-test with a significance level of *p* < 0.01 to compare results between pairs of treatments. The symbol (*) indicates significant differences between treatments. Fluorescence images show individual microspheres.

In our case, we continuously perfused 5-FU for 46 h in a well-mixed system, followed by perfusion with culture medium without 5-FU for three more days to mimic the progressive clearance of the drug from blood circulation. We believe that our results could be more easily translated into a real cancer treatment due to the dynamic conditions in this ToC, that combine convective and diffusive transport and enable the recreation of different therapeutic regimes in terms of overall perfusion times, doses, and overall residence times. Our results also showed that Caco2 cells start to decay after two days of drug perfusion, as observed by a tendency toward a decrease in glucose consumption (Figure 5B). Glucose consumption appeared to increase 24 h after drug perfusion, but it later decreased again, a behavior that has been previously observed [45]. Studies have shown that cancer cells respond to chemotherapeutic drugs by rewiring their metabolism as a mechanism of adaptive resistance [46]. Some enzymes of the glycolytic pathway are upregulated in numerous cancer cells [47]. In this case, our Caco2 cells may have increased their metabolism in response to treatment with 5-FU, but the dose was ultimately high enough to compromise cell viability.

We also evaluated the expression of two relevant biomarkers (VEGF-A and E-cad) in the anti-cancer assays using 5-FU. The co-expression of VEGF-A is reported to be highly correlated with tumor resistance to first-line therapy with several drugs, such as irinotecan, fluorouracil, and leucovorin [48]. Independently, each of these markers is also significant in colorectal cancer progression. VEGF is a proangiogenic factor that binds to sites on endothelial cells to promote proliferation, migration, and vascular permeability [49,50], and it is overexpressed in diverse types of cancer, including colorectal cancer [51]. Previous preclinical and clinical studies have demonstrated that VEGF is the main angiogenic factor in human colorectal cancer and is associated with metastatic events and poor patient prognosis [17]. In addition, an increase has been observed in VEGF expression in engineered microtumors grown in 3D systems [52,53,54]. Caco2 cells may express VEGF-A during normoxia, but VEG-AF expression can be increased several-fold during hypoxia [55]. Consistently, our experiments showed that VEGF-A expression in our tumor-on-chip system increased by 8-fold from day 0 to day 10 in regular culture medium conditions (Figure 5C). 

We embedded Caco2 microtumors in spheres with diameters larger than 400 μm, which exceeded the maximum distance to which cells can effectively receive oxygen and nutrients (approximately 200 μm, as observed in real avascular tumors) [56]. Thus, the size of our hydrogel microspheres favors the formation of hypoxic cores. The oxygen gradient formed from the center to the periphery of the microspheres would explain the increase in VEGF-A expression of Caco2 microtumors. By contrast, VEGF-A expression in Caco2 microtumors dropped to almost zero after exposure to 5-FU. This is consistent with the expected and previously mentioned DNA and RNA disruption caused by this drug in cancer cells [57]. When exposed to DMSO (vehicle control), VEGF-A expression showed a 5-fold increment (Figure 5C). The recreation of hypoxic cores is relevant in cancer models for different reasons. For example, previous reports have stated that cancer stem cells are able to adapt to hypoxia; therefore, they develop resistance to treatments such as chemotherapy and radiotherapy in hypoxic environments [58]. Our ToC model may be more accurate for testing different drugs, as it recapitulates the hypoxic conditions seen in vivo.

E-cad is a fundamental glycoprotein required for intercellular adhesion that also acts as a suppressor gene for tumor growth and progression [59]. It is viewed as an effective biomarker for monitoring patients with colorectal cancer [60].

Experimental evidence has shown that colorectal cancer cells that express E-cad are more resistant to chemotherapeutic treatments [59], because E-cad prevents drug diffusion through the intercellular junctions [60]. E-cad has also been identified as a key marker in the epithelial-to-mesenchymal transition, a process during which adherence junctions (such as E-cad, and others) are dismantled [60]. Consequently, this leads to the loss or aberrant expression of E-cad, a key feature of the EMT. In our experiments, E-cad expression on day 10 was significantly lower after treatment with both 5-FU and DMSO than in the pristine controls (i.e., system perfused with culture medium only). This decrease in E-cad expression could also be closely related to the observed sensitivity to the chemotherapeutic treatment with 5-FU. By contrast, no significant decrease was evident in the pristine control between days 0 and 10 (Figure 5D). 

Our results demonstrate that our chips are suitable for the culture and maturation of colon cancer microtumors for studying cancer biology or for drug testing, and that our findings are consistent with previously published research [25,45].

## 4. Conclusions

We developed and characterized a millifluidic device that enables the culture of cancer microtissues embedded in alginate/GelMA microbeads. This ToC is simple to fabricate, assemble, and use, and its rhomboidal culture chamber can hold relatively large volumes of microtissues (i.e., in the order of 0.5–1 cm^3^). Our mixing and transport characterization experiments suggest that this ToC approach can recreate the convective/diffusive transport of nutrients and anti-cancer drugs that occurs in real tumor niches, where compounds are first convectively transported by the blood to the tissues to finally reach cells by diffusion. 

In a first set of experiments, we analyzed the convective and diffusive transport inside the rhomboidal culture chamber using a fluorescent tracer (FITC-dextran). Comparison of the dispersion of FITC-dextran within the culture chamber when empty, when occupied with a solid piece of hydrogel with a central channel, and when filled with an array of hydrogel microbeads revealed that the most effective convective and diffusive transport was achieved with a chamber filled with the GelMA/alginate microspheres.

Use of this 3D-printed chip to culture Caco2 cell-laden microbeads for several days confirmed the feasibility of extended cell culture on-chip via the formation of cell aggregates on-chip that reached a relatively steady glucose consumption after 5 days of culture. Exposure of these Caco2 aggregates to 5-FU in on-chip drug assays that emulated the residence time, dose, and duration of typical 5-FU chemotherapy resulted in an increase in dead cells and a decrease in cell metabolism, in agreement with other reports of colorectal ToCs. We also observed an increased expression of VEGF in culture from day 0 to day 10, indicating the induction of hypoxic conditions at the center cores of our large-sized (>400 μm diameter) hydrogel microspheres. Similar hypoxic conditions are observed at the onset of the development of real avascular tumors in cancer patients. We also observed decreased expression of VEGF after 5-FU treatment, as expected due to the disruption of DNA and RNA synthesis caused by this drug. Measurement of E-cad expression, which has been related to resistance to chemotherapeutic agents, revealed decreased E-cad expression in our on-chip experiments upon exposure of the microspheres to 5-FU.

In sum, we have introduced a simple 3D-printed tumor-on-chip system suitable for use as a platform for drug testing and studying cancer biology. Our results also illustrate that the combination of 3D biofabrication and tumor-on-chip platforms might further our understanding of the effects of different treatments used in colorectal and other types of cancer in ToCs cultured in relatively simple 3D-printed bioreactors. A limited number of examples of 3D-printed ToCs (and organs-on-chips) can be found in the literature [10,61]. More work must be done on this topic to demonstrate the multiple advantages of 3D-printed cell and tissue culture systems, in terms of flexibility of design (i.e., freedom in terms of configurations and rapid prototyping iterations) and practicality of fabrication and use (i.e., monolithic structures that reduce leakage and facilitate operation). Here, we introduced a 3D-printed ToC that is much larger than other 3D-printed culture devices previously presented. The ability to culture larger cancer tissues provides practical benefits, including a greater yield of biological material for post-culture analysis (i.e., live/dead staining, immunostaining, and qPCR). Arguably, larger continuous chips could be more representative of clinically relevant tumors than most microfluidic chips, as real tumors are typically much larger in size than microfluidic-based ToCs. The transport characterization that we conducted on-chip is another innovative aspect of this work. Our experiments demonstrate that the simple ToC platform introduced here fosters effective convective and well-mixed conditions in the liquid niche while enabling the recapitulation of diffusive transport from the well-mixed liquid niche to the cell-laden hydrogel microbeads. This is relevant for recapitulating not just tumor scenarios, but many other tissue niches.

## Figures and Tables

**Table 1:** Primer sequences for RT-qPCR.

Gene	Forward Primer	Reverse Primer
VEGF-A	CACCATCGACAGAACAGTCC	GAATCCAATTCCAAGAGGGA
E-cadherin	CCCGCCTTATGATTCTCTGCTCGTG	TCCGTACATGTCAGCCAGCTTCTTG
GAPDH	GAGTCAACGGATTTGGTCG	TTGATTTTGGAGGGATCTCG

## Data Availability

The data presented in this study are available in this article and its supplementary materials.

## Supplementary Materials

The following supporting information can be downloaded at: https://www.mdpi.com/article/10.3390/bioengineering10050554/s1, Figure S1: Image analysis for fluorescence measurements; Figure S2: Comparison of culture media diffusion in two different mini-reactor geometries. Figure S3: Survival of Caco2 microtumors in different regions of the ToC.
