# Supplementary material for: 3D-Printed Tumor-on-Chip for the Culture of Colorectal Cancer Microspheres: Mass Transport Characterization and Anti-Cancer Drug Assays
Source: Bioengineering (Basel). 2023 May 5;10(5):554. doi: 10.3390/bioengineering10050554 (PMC10215397; doi:10.3390/bioengineering10050554)
Supplement: Supplementary file 1 [file bioengineering-10-00554-s001.zip › Supplementary Figures.pdf]

Supplementary Figures

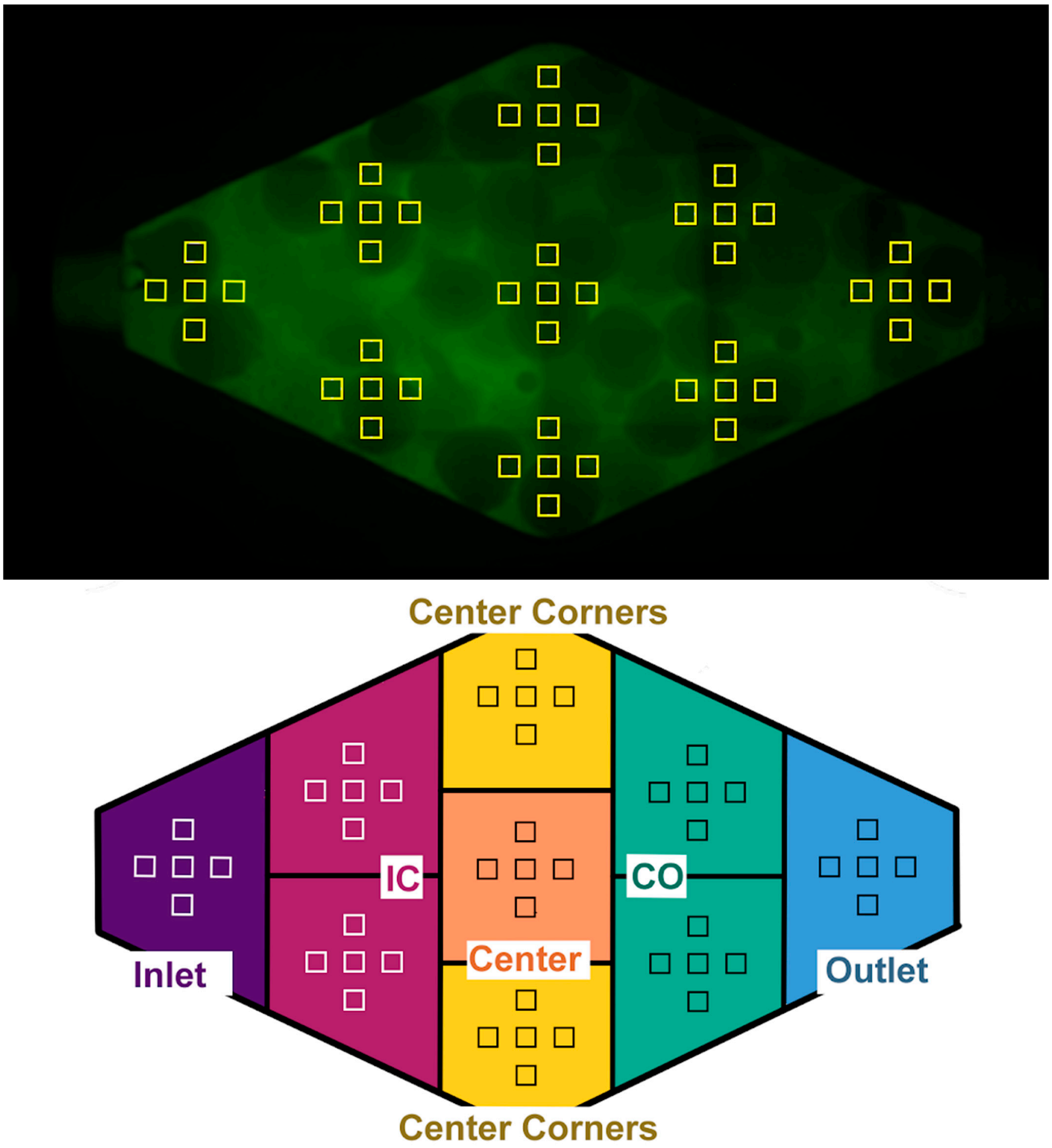

Figure S1. Image analysis for fluorescence measurements. Mean pixel fluorescence was obtained in each of the five 45000 pixel^2 and divided by area. We obtained the data per each hour in a 14-hour experiment. Due to the ToC's symmetry and similar numerical results, the mean was obtained for the center corners, IC and CO to be better displayed.

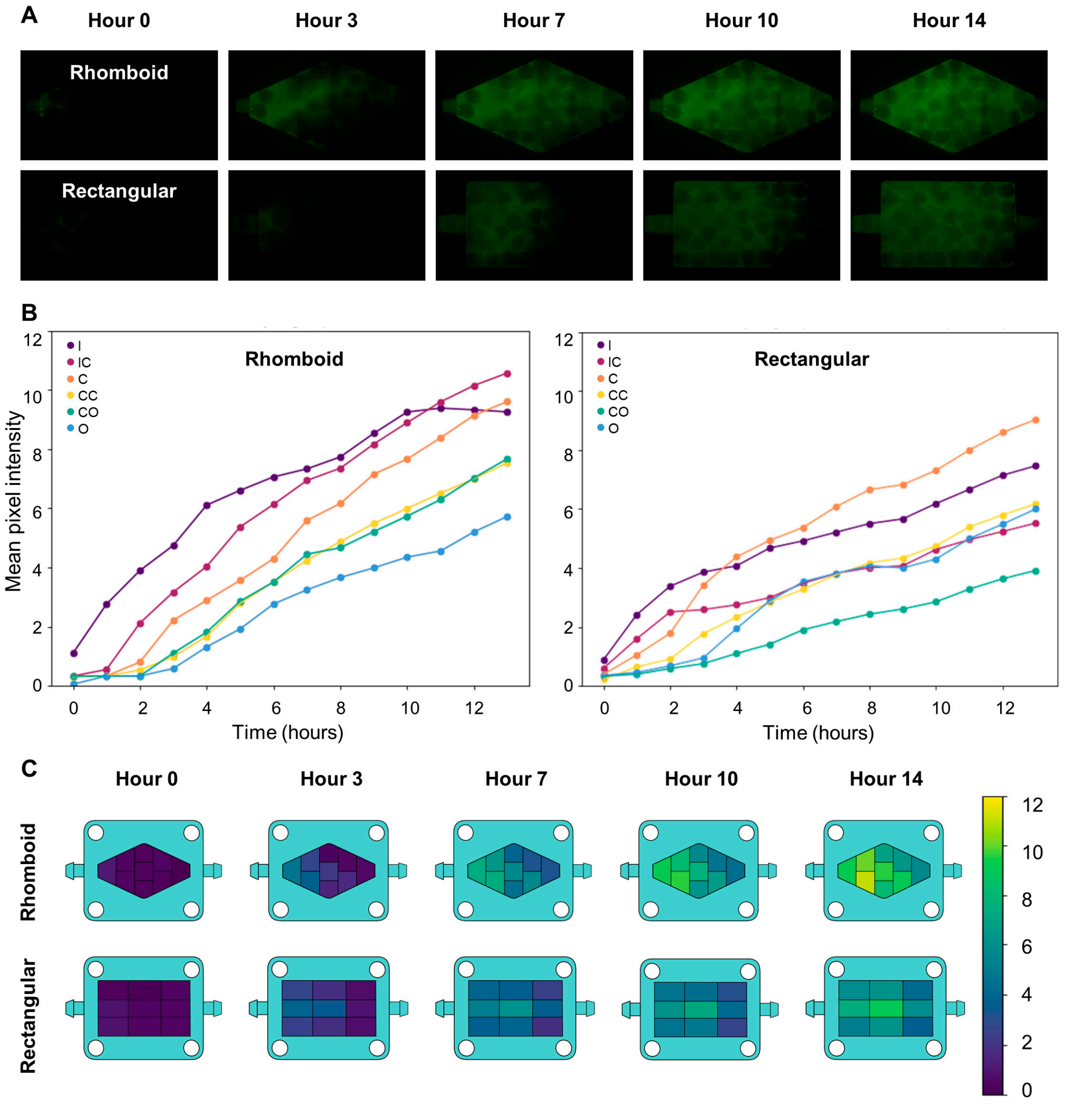

Figure S2. Comparison of culture media diffusion in two different ToC geometries. A) Fluorescence intensity change observed with FITC-dextran inside the mini-reactor in two different pool geometries: rhomboid and rectangular with 3.5% Alg + 2.5% GelMA microspheres. B) Quantification and comparison of culture media diffusion in the two different settings as represented by the increase in fluorescence intensity (FITC-dextran), measured by mean pixel intensity. C) Heatmaps representing culture media diffusion throughout the mini-reactor in different zones at several time points as observed by the increase in fluorescence.

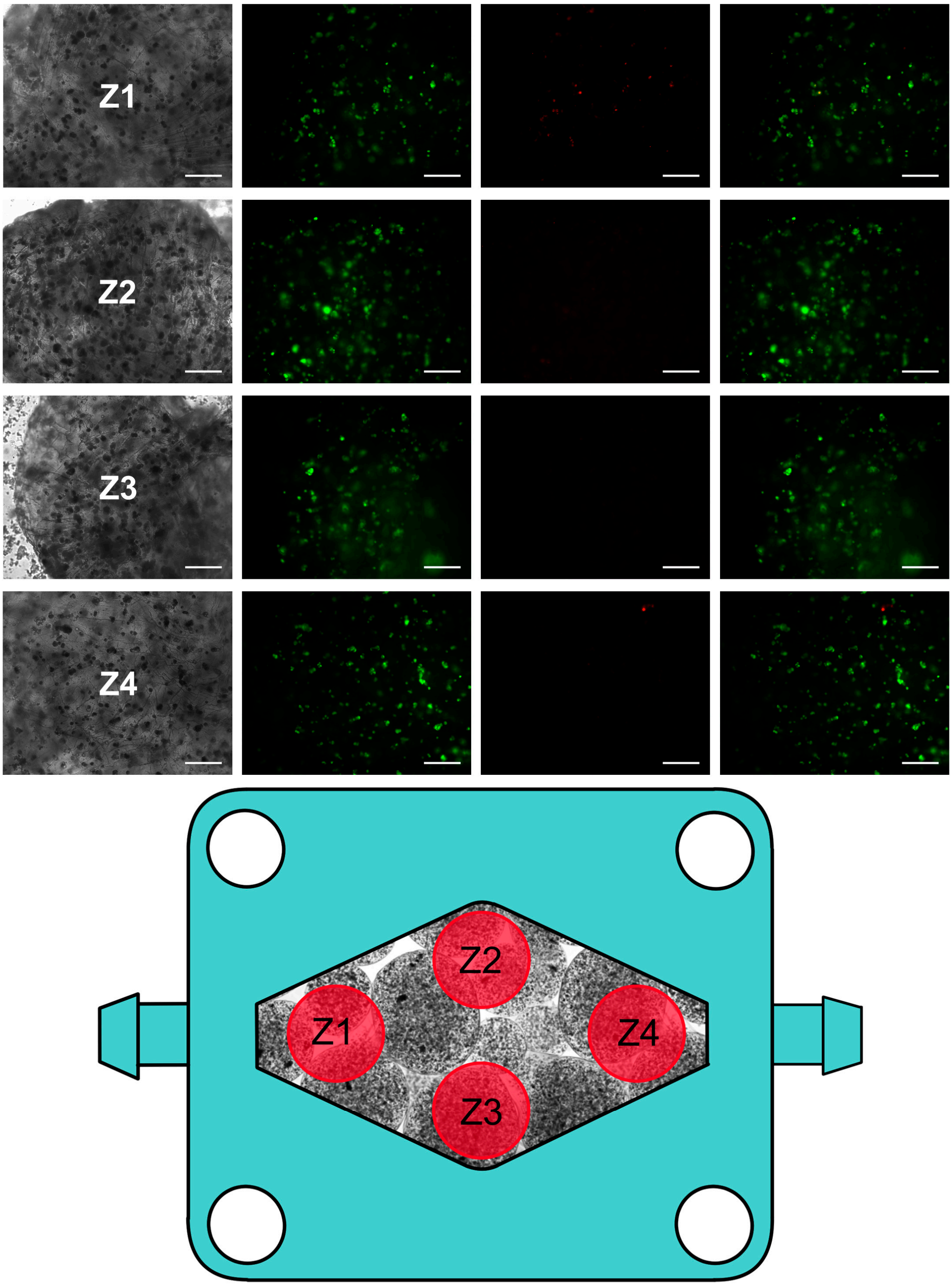

Figure S3. Survival of Caco2 microtumors in different regions of the ToC. Top: Bright field, Green channel (live), Red channel (dead), and merged fluorescence channels images of microspheres retrieved from different regions of the ToC. Scale bar: 200 µm. Bottom: Map of the regions from which the microspheres were retrieved.
